# Supplementary material for: The Impact of Environmental Habitats and Diets on the Gut Microbiota Diversity of True Bugs (Hemiptera: Heteroptera)
Source: Biology (Basel). 2022 Jul 11;11(7):1039. doi: 10.3390/biology11071039 (PMC9312191; doi:10.3390/biology11071039)
Supplement: Supplementary file 1 [file biology-11-01039-s001.zip › Supplementary Figure S1.The representative bacterial genera associated with different heteropteran families identified based on linear discriminant analysis effect size.pdf]

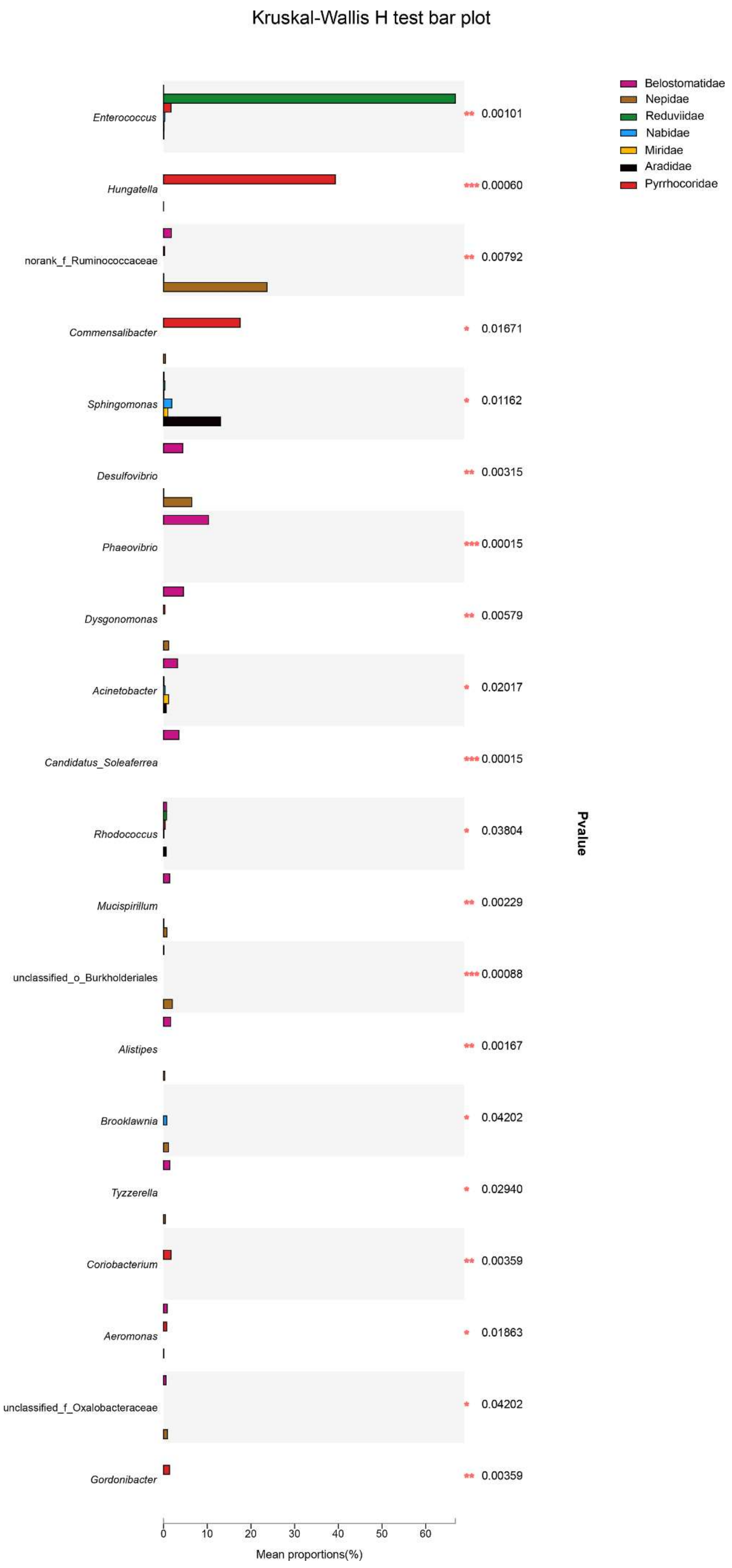

Supplementary Figure S1. The representative bacterial genera associated with different heteropteran families identified based on linear discriminant analysis effect size (LEfSe). No representative bacterial genus of Notonectidae was determined due to the small specimen number.
